# Supplementary material for: Functional Enrichment Analysis of Regulatory Elements
Source: Biomedicines. 2022 Mar 3;10(3):590. doi: 10.3390/biomedicines10030590 (PMC8945021; doi:10.3390/biomedicines10030590)
Supplement: Supplementary file 1 [file biomedicines-10-00590-s001.zip › FileS2.pdf]

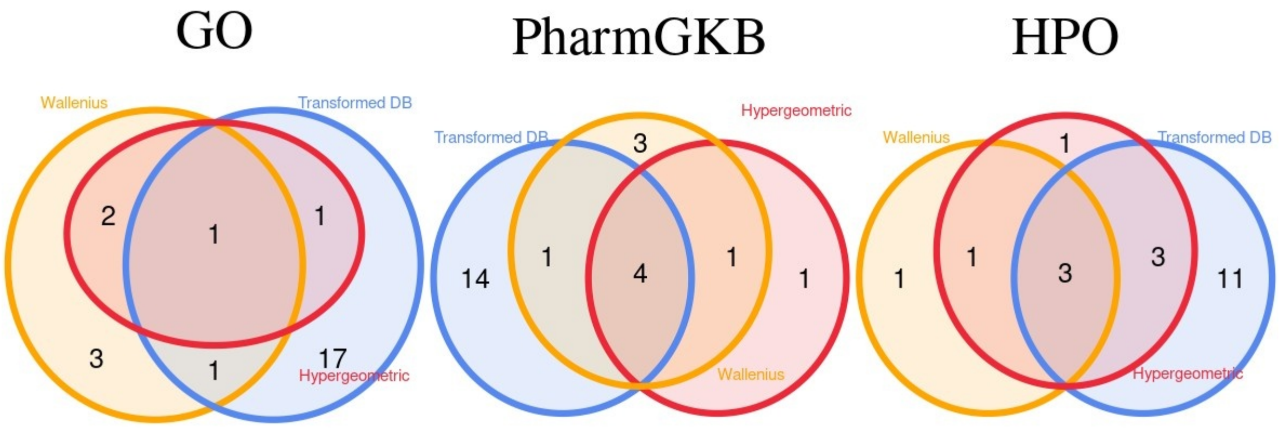

Figure S1. Venn diagrams of the overlap between the heart related terms found in the top 20 enriched terms counted in Table 1 for each annotation and strategy: Wallenius in yellow, Transformed database (DB) in blue and the standard Hypergeometric in red.

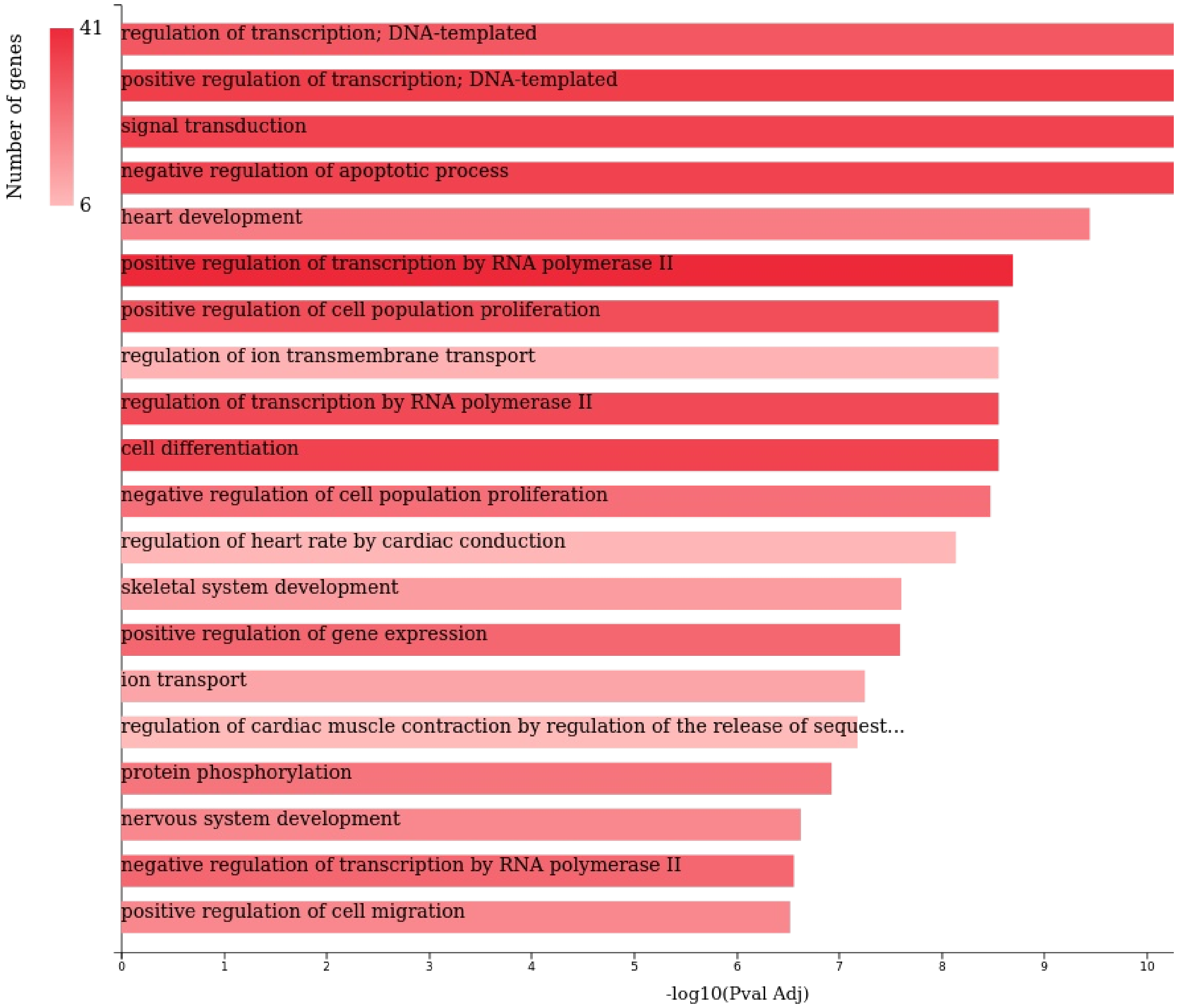

Figure S2. Bars chart with the top 20 terms found with the Wallenius approach and the Gene Ontology Biological Process database. Plot based in the results found in the sheet 1 (S.Table.1-Wallenius-GOBP) of supplementary excel File S1.

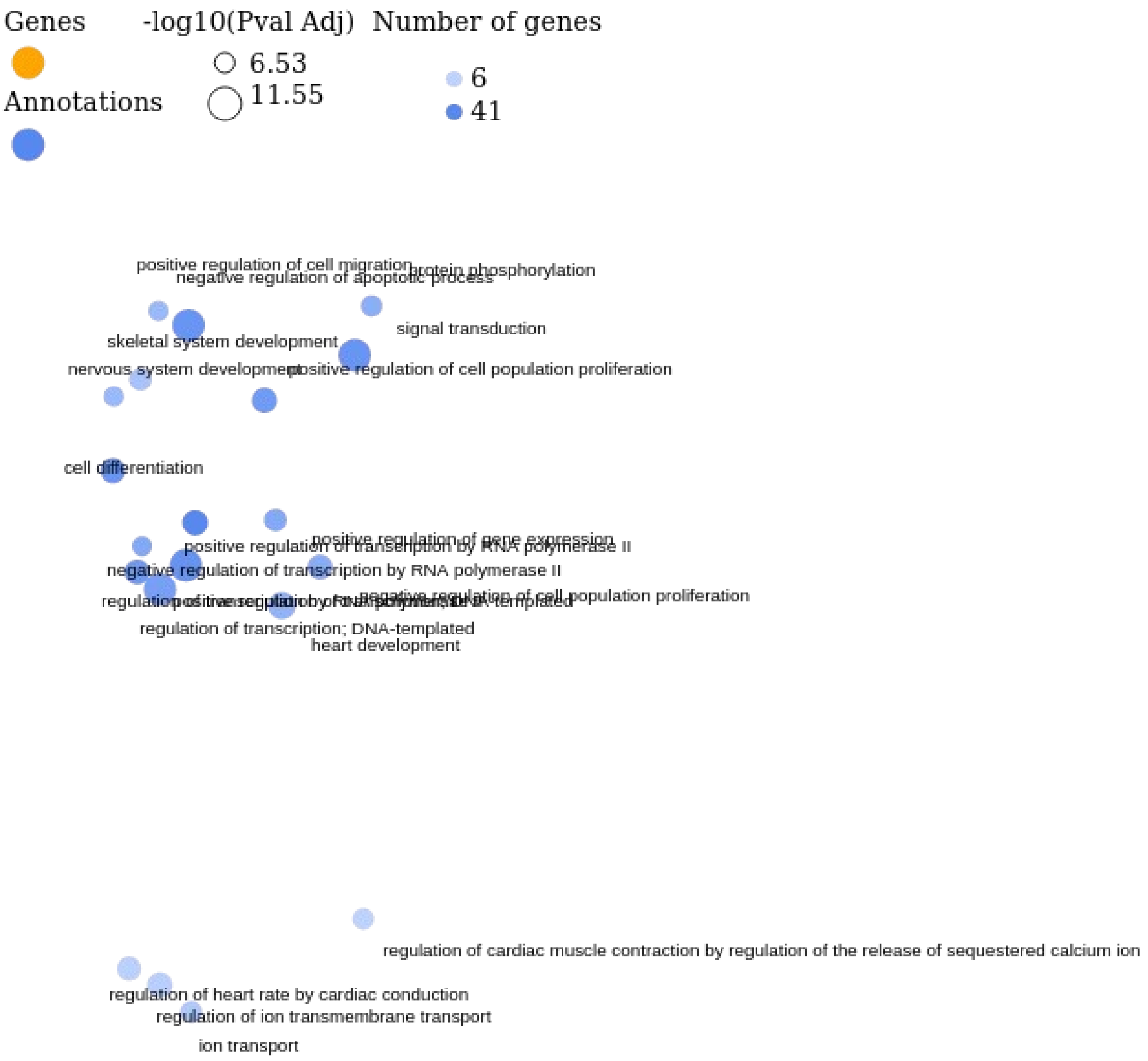

Figure S3. Network plot of gene and annotations but with the genes hidden of the top 20 terms found with the Wallenius approach and the Gene Ontology Biological Process database. Plot based in the results found in the sheet 1 (S.Table.1-Wallenius-GOBP) of supplementary excel File S1.

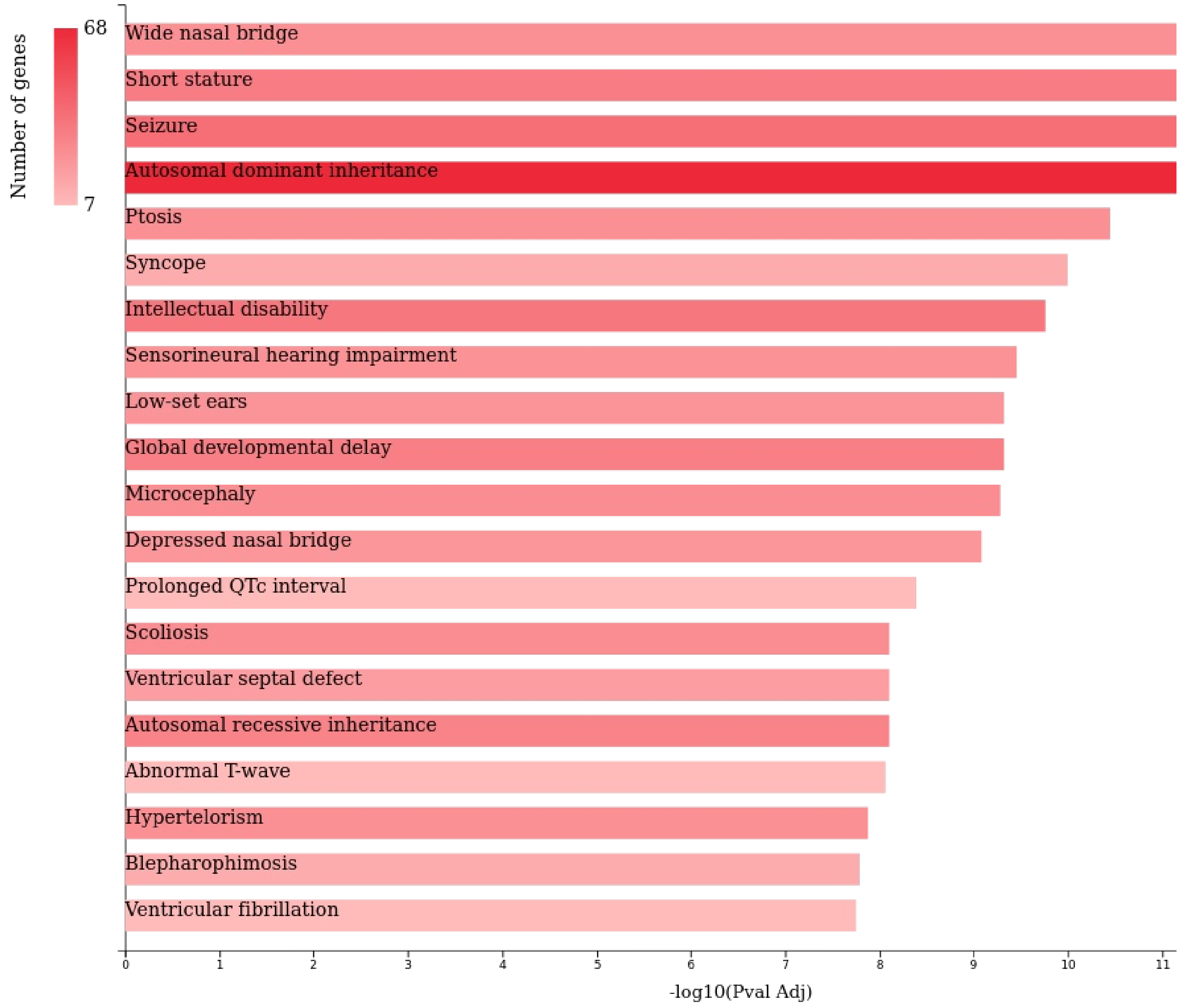

Figure S4. Bars chart with the top 20 terms found with the Wallenius approach and the Human Phenotype Ontology database. Plot based in the results found in the sheet 2 (S.Table.2-Wallenius-HPO) of supplementary excel File S1.

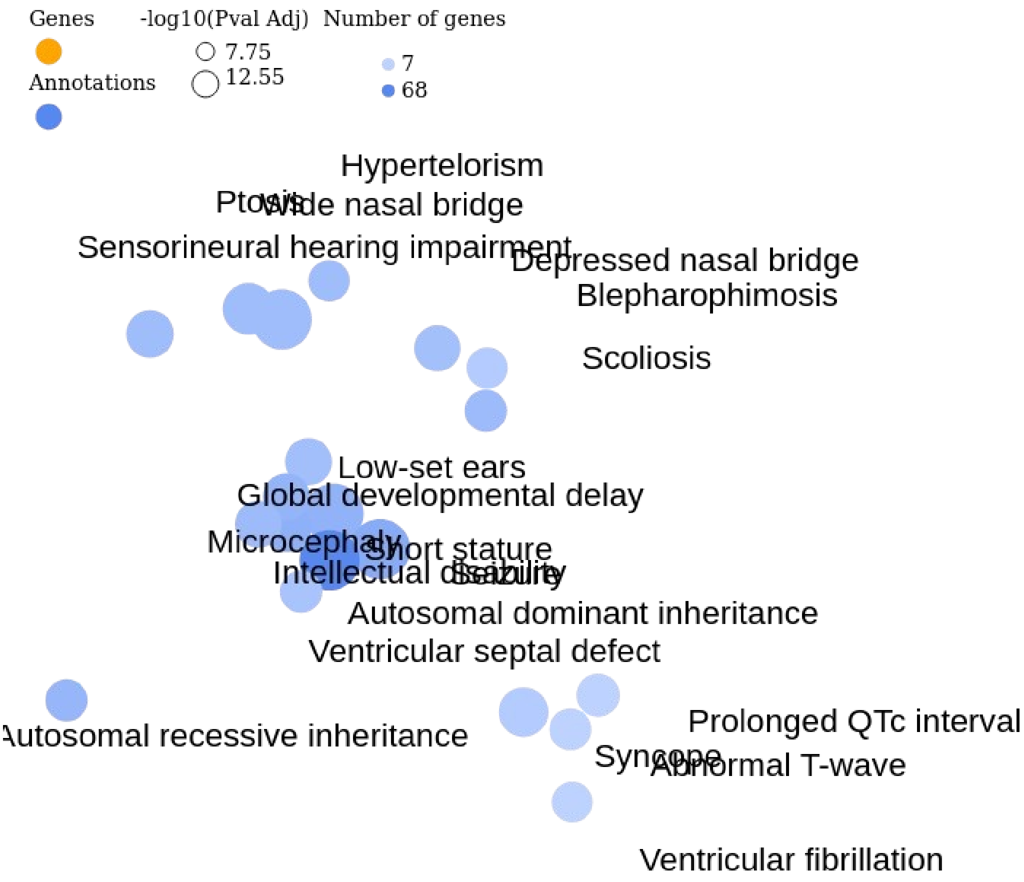

Figure S5. Network plot of gene and annotations but with the genes hidden of the top 20 terms found with the Wallenius approach and the Human Phenotype Ontology database. Plot based in the results found in the sheet 2 (S.Table.2-Wallenius-HPO) of supplementary excel File S1.

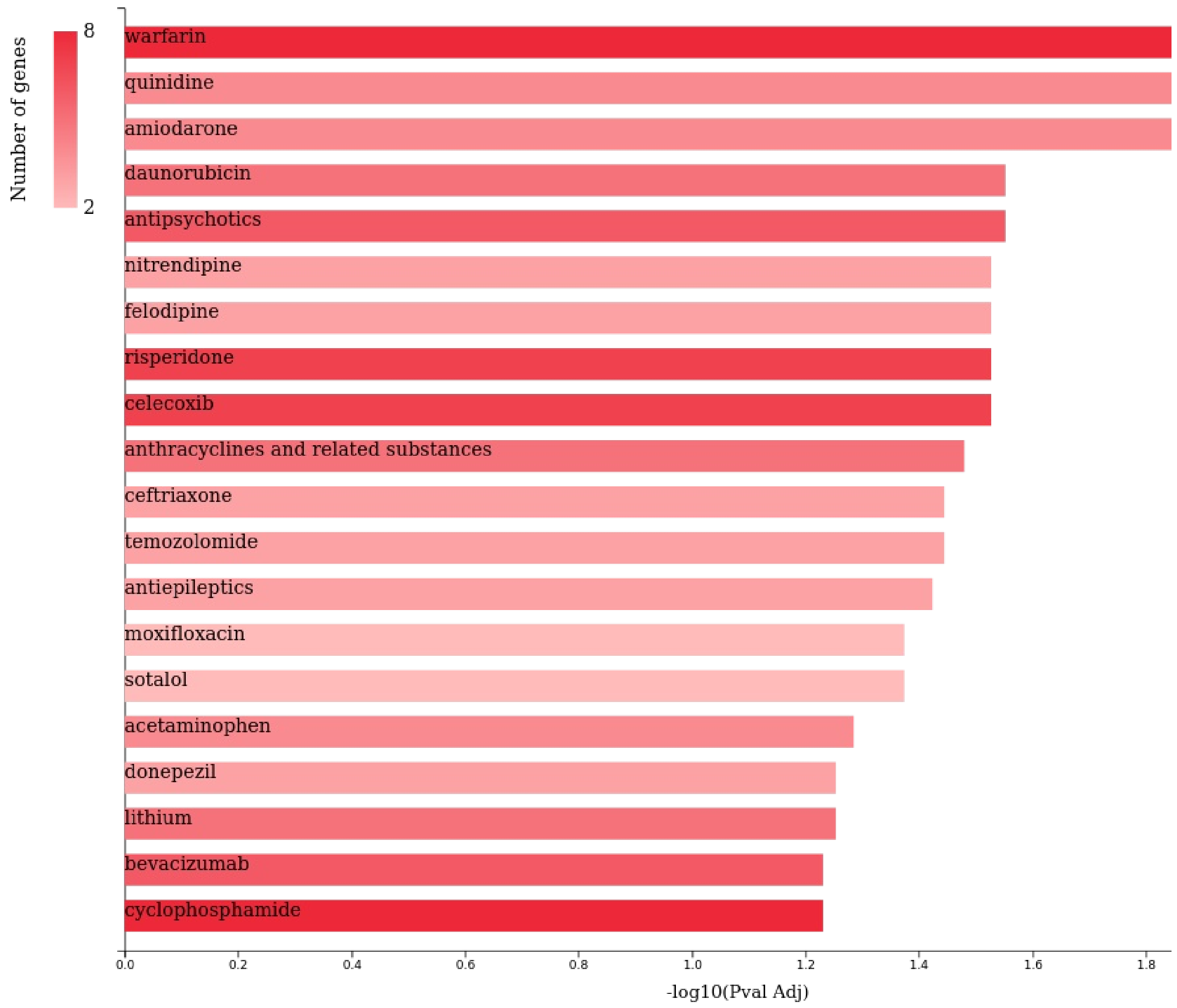

Figure S6. Bars chart with the top 20 terms found with the Wallenius approach and the PharmGKB database. Plot based in the results found in the sheet 3 (S.Table.3-Wallenius-PharmGKB) of supplementary excel File S1.

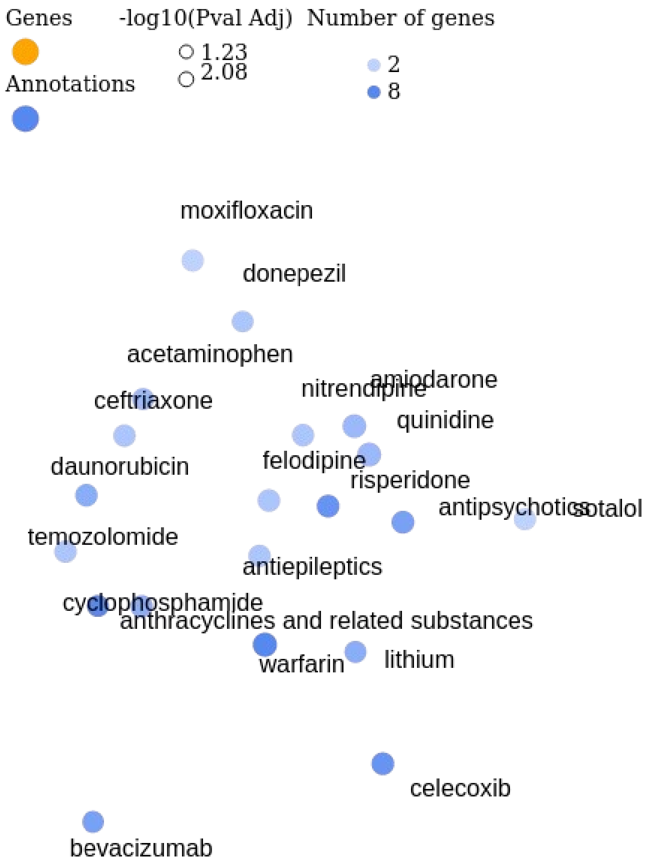

Figure S7. Network plot of gene and annotations but with the genes hidden of the top 20 terms found with the Wallenius approach and the PharmGKB database. Plot based in the results found in the sheet 3 (S.Table.3-Wallenius-PharmGKB) of supplementary excel File S1.

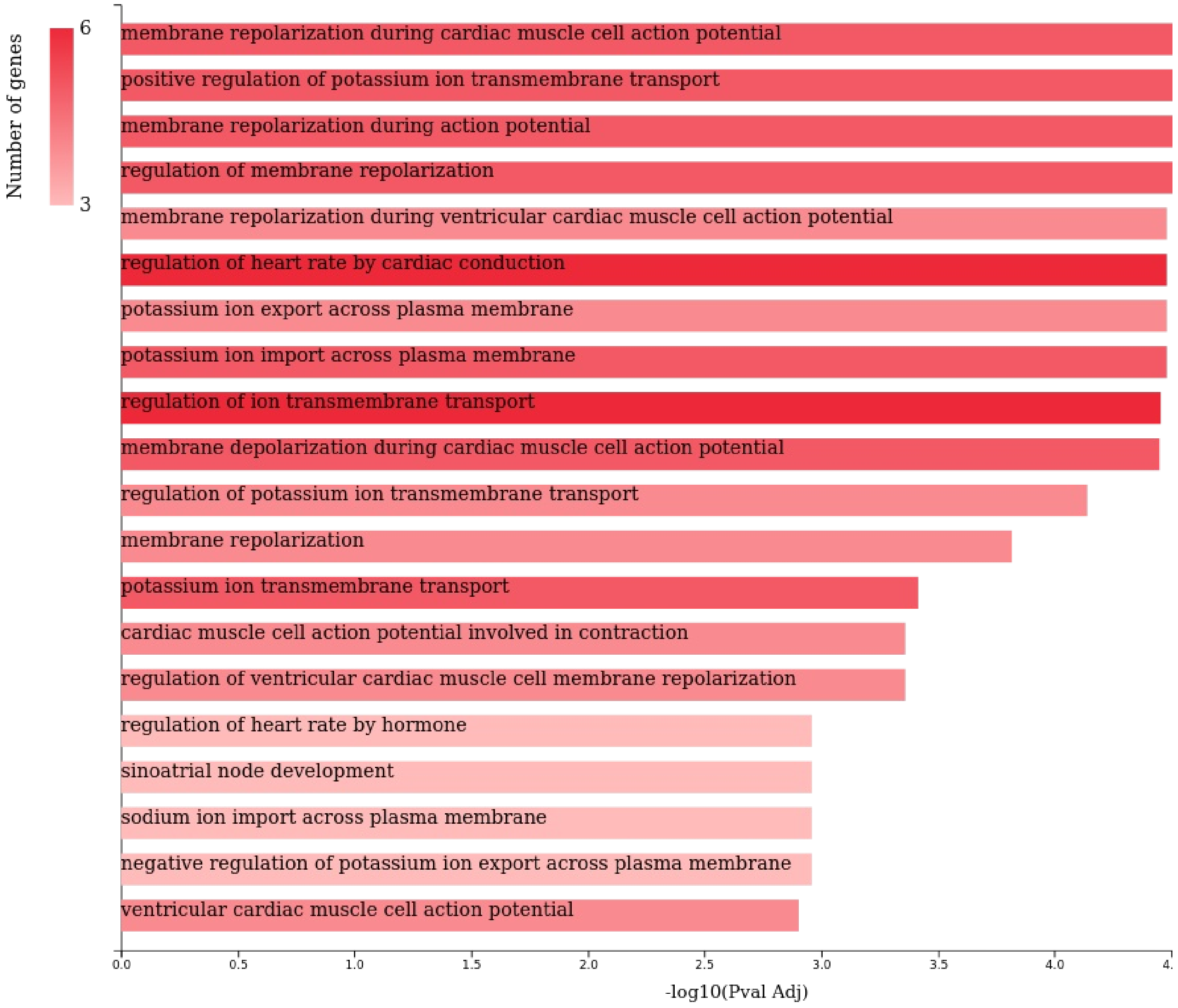

Figure S8. Bars chart with the top 20 terms found with the transformed database approach and the Gene Ontology Biological Process database. Plot based in the results found in the sheet 4 (S.Table.4-TransformedDB-GOBP) of supplementary excel File S1.

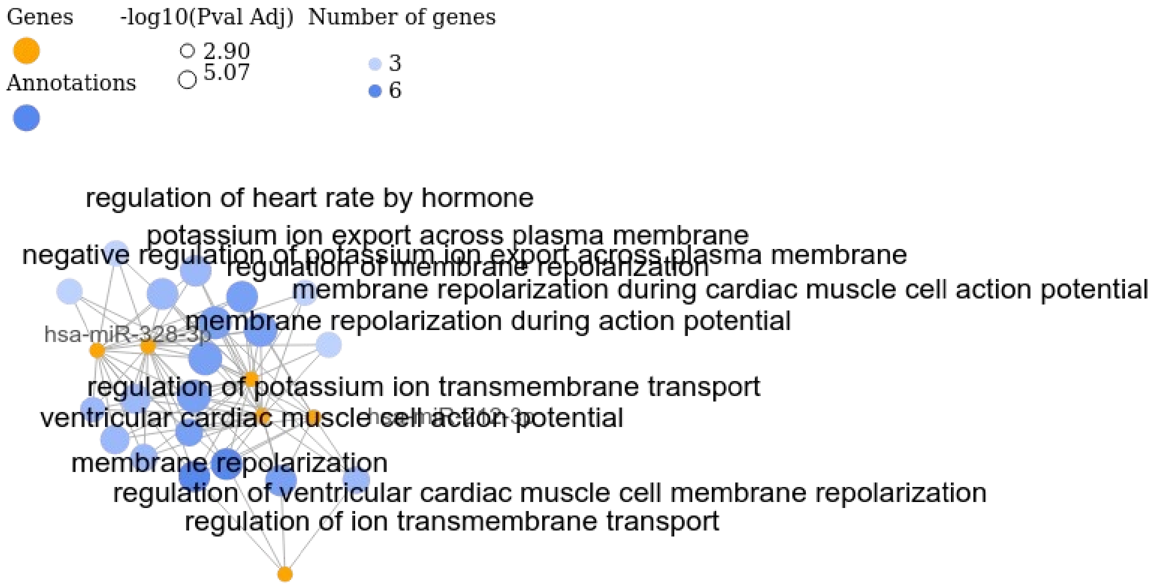

Figure S9. Network plot of miRNAs and its annotations of the top 20 terms found with the transformed

database approach and the Gene Ontology Biological Process database. Plot based in the results found in the sheet 4 (S.Table.4-TransformedDB-GOBP) of supplementary excel File S1.

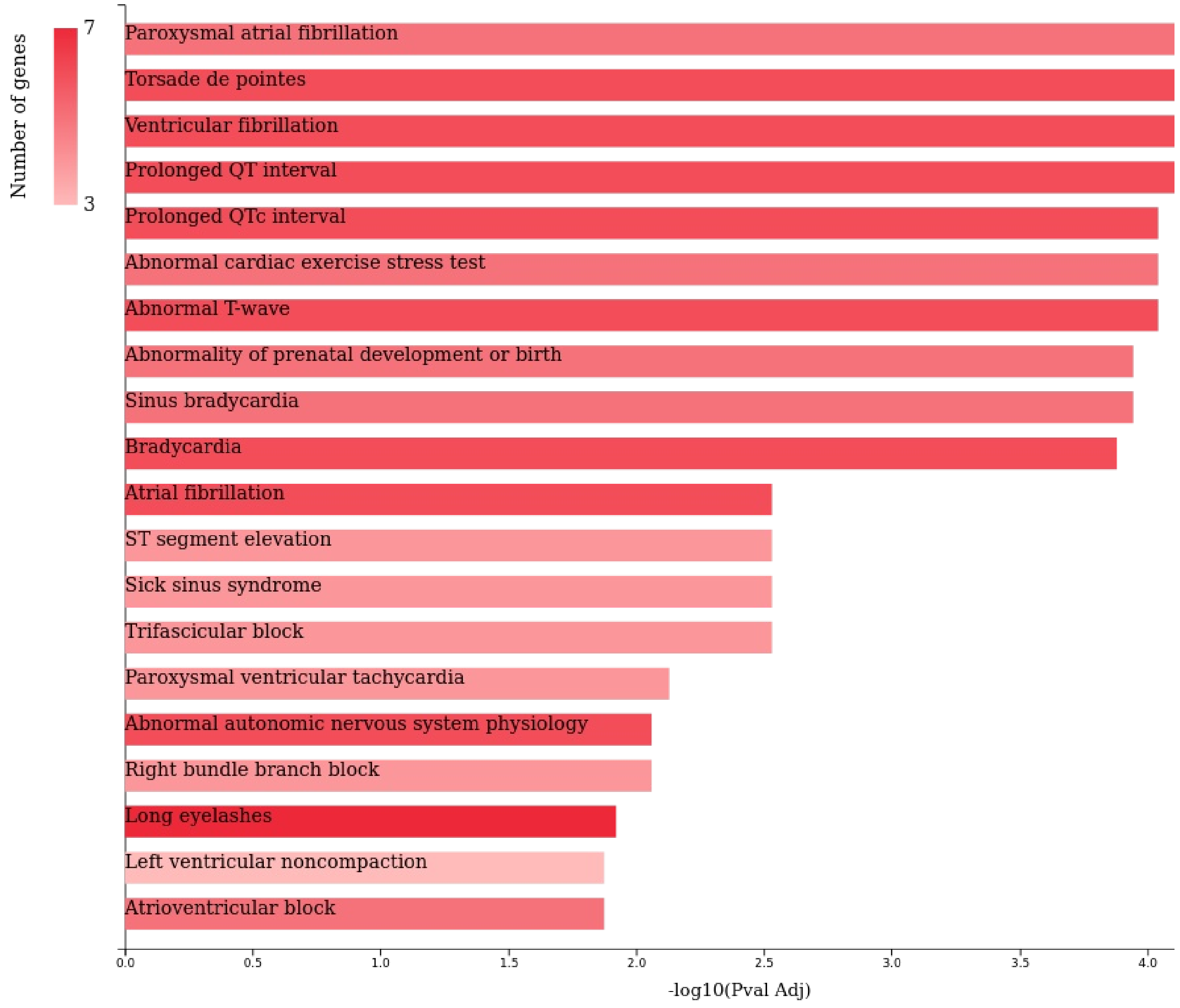

Figure S10. Bars chart with the top 20 terms found with the transformed database approach and the Human Phenotype Ontology database. Plot based in the results found in the sheet 5 (S.Table.5-TransformedDB-HPO) of supplementary excel File S1.

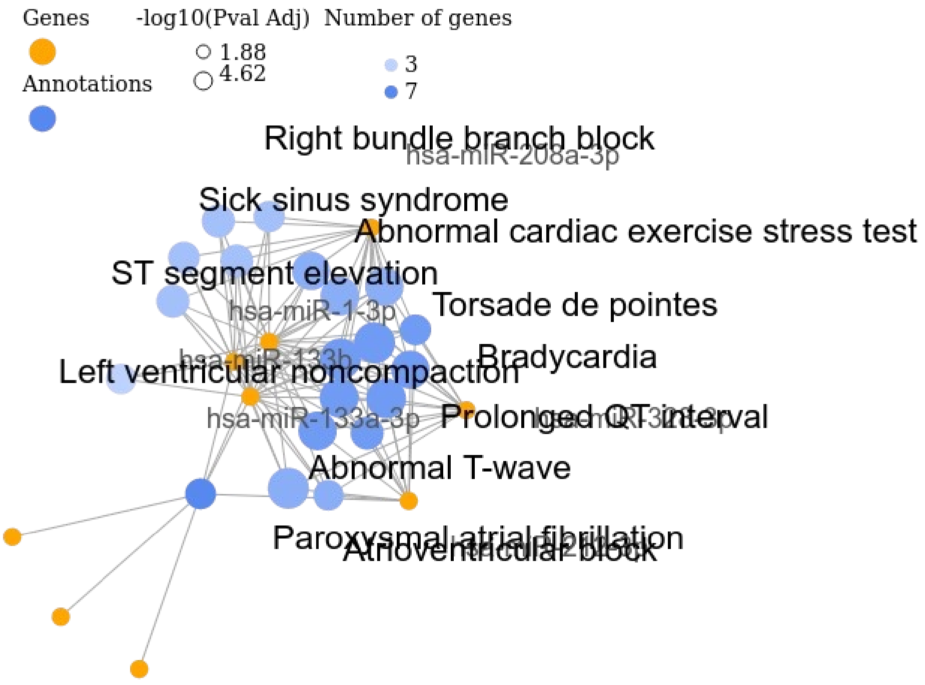

Figure S11. Network plot of miRNAs and its annotations of the top 20 terms found with the transformed database approach and the Human Phenotype Ontology database. Plot based in the results found in the sheet 5 (S.Table.5-TransformedDB-HPO) of supplementary excel File S1.

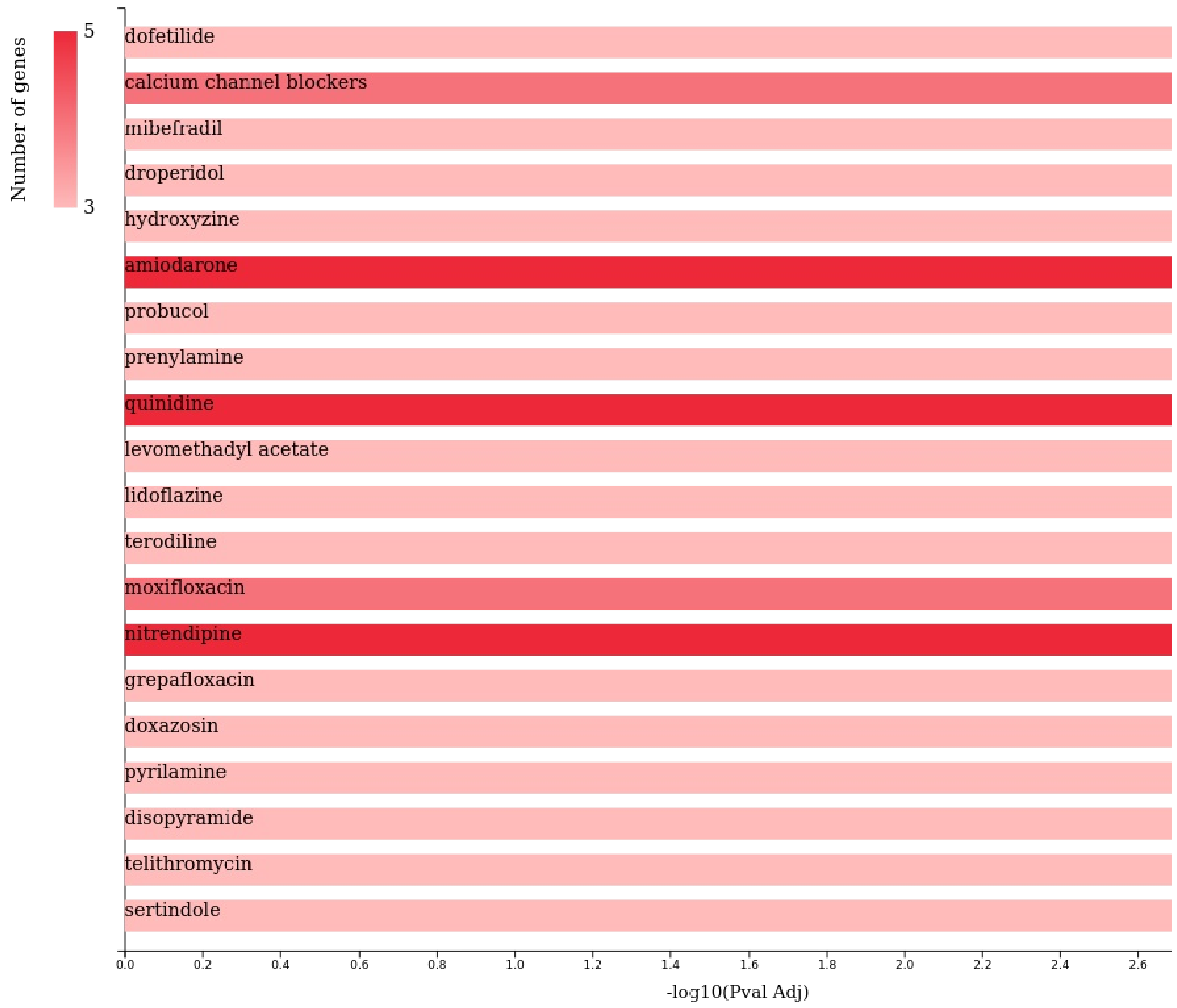

Figure S12. Bars chart with the top 20 terms found with the transformed database approach and the PharmGKB database. Plot based in the results found in the sheet 6 (S.Table.6-TransformedDB-PharmGKB) of supplementary excel File S1.

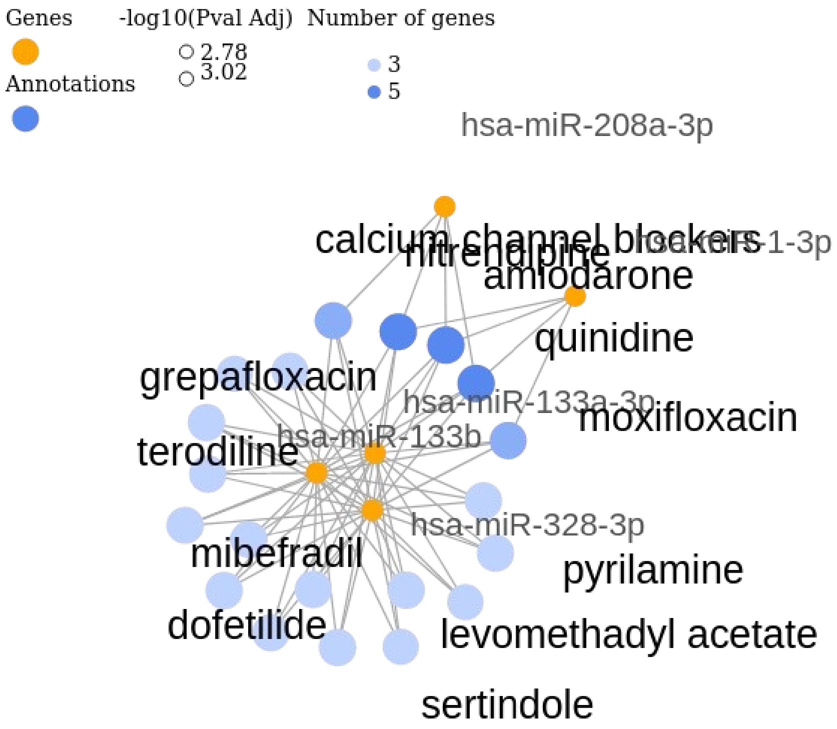

Figure S13. Network plot of miRNAs and its annotations of the top 20 terms found with the transformed database approach and the PharmGKB database. Plot based in the results found in the sheet 6 (S.Table.6-TransformedDB-PharmGKB) of supplementary excel File S1.

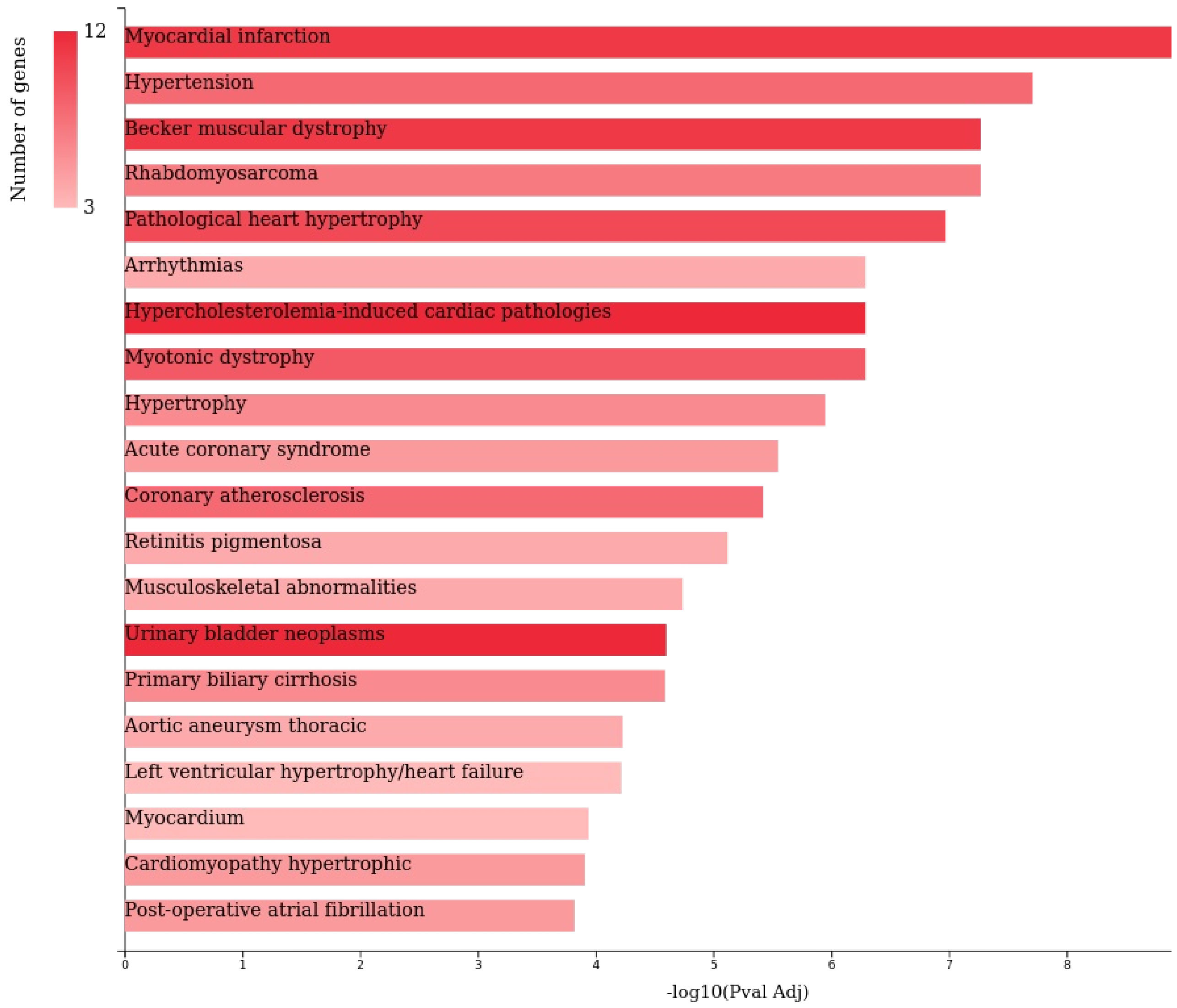

Figure S14. Bars chart of the top 20 terms found with the approach based on the use of databases with direct annotation of miRNAs, the Mammalian ncRNA-Disease Repository in this case. Plot based in the results found in the sheet 7 (S.Table.7-MNDR) of supplementary excel File S1.

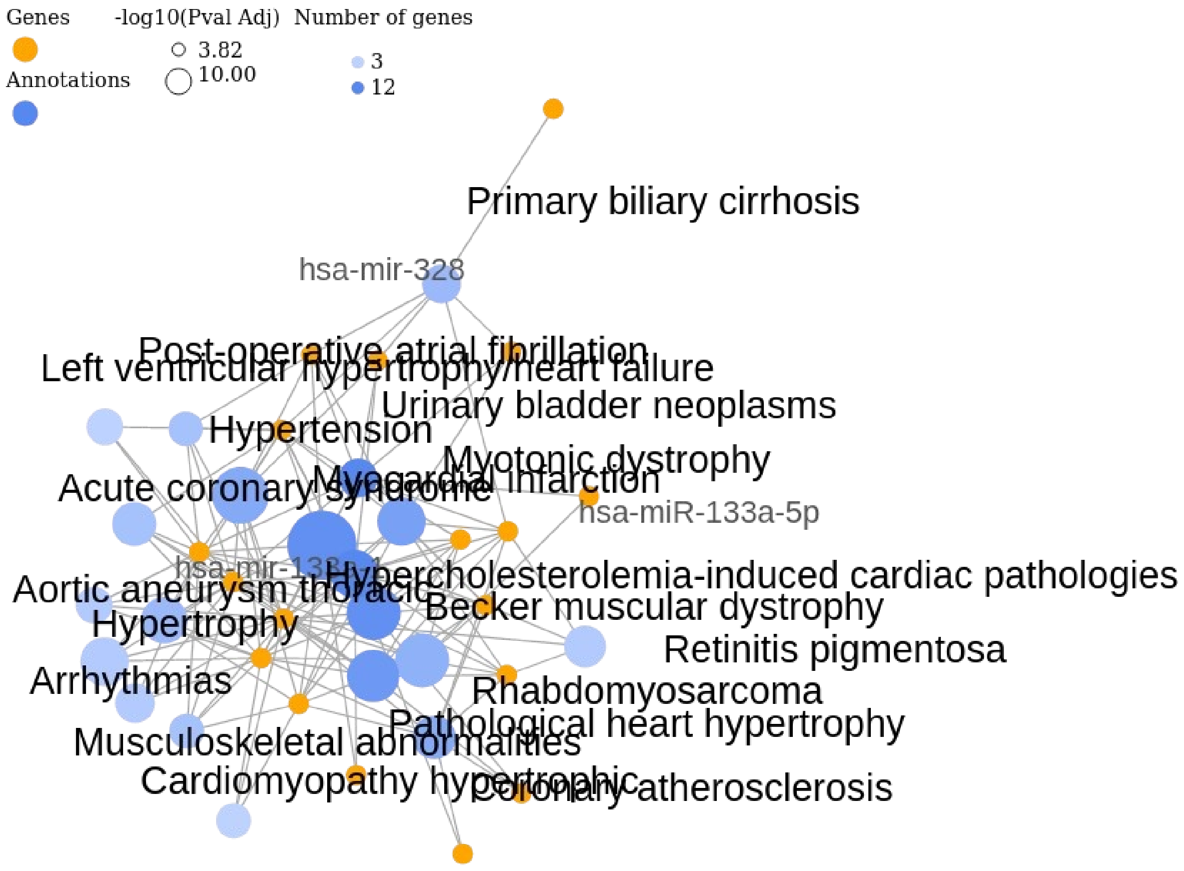

Figure S15. Network plot of miRNAs and its annotations of the top 20 terms found with the direct annotation of miRNAs databases, the Mammalian ncRNA-Disease Repository in this case. Plot based in the results found in the sheet 7 (S.Table.7-MNDR) of supplementary excel File S1.

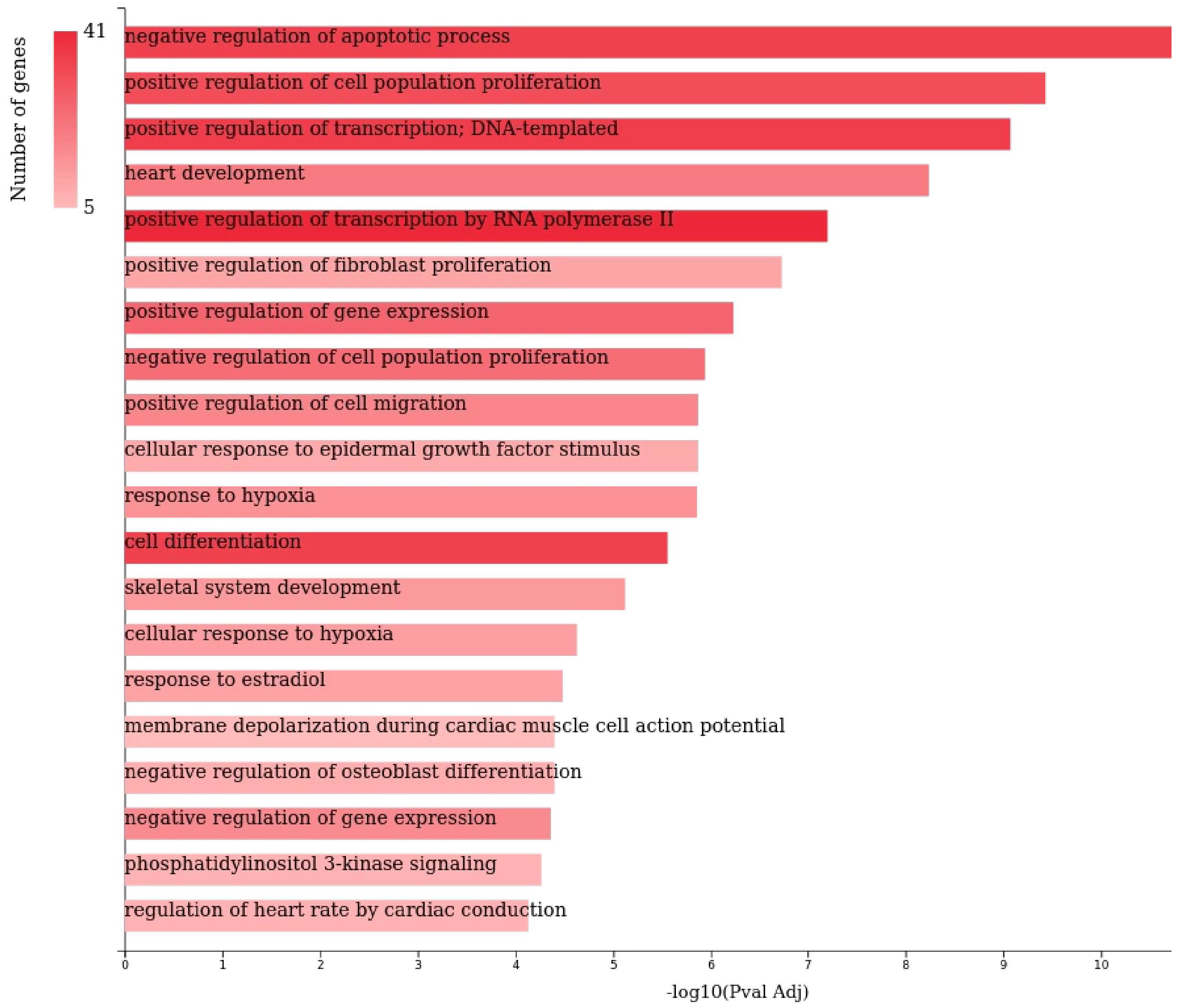

Figure S16. Bars chart with the top 20 terms found with the standard Hypergeometric approach and Gene Ontology Biological Process database. Plot based in the results found in the sheet 8 (S.Table.8-Hypergeometric-GOBP) of supplementary excel File S1.

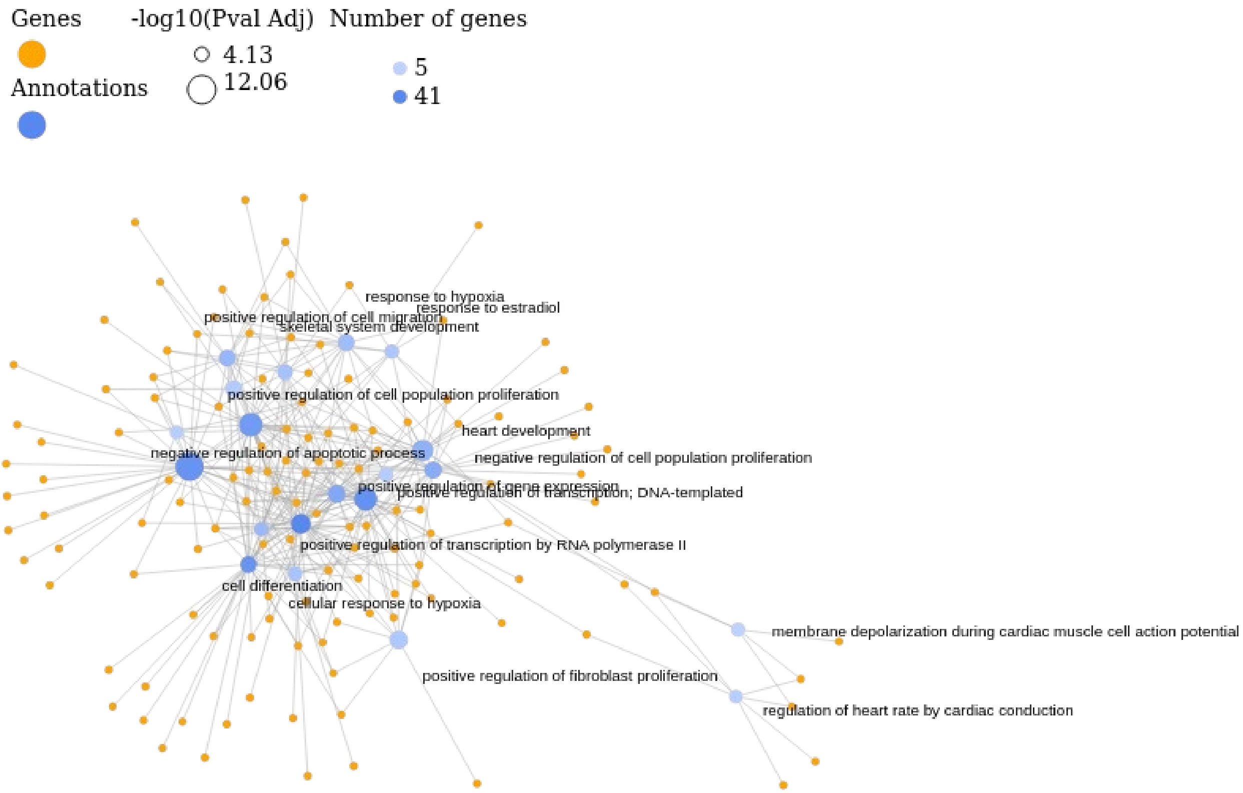

Figure S17. Network plot of gene, with their label hidden, and annotations of the top 20 terms found with the standard Hypergeometric approach and Gene Ontology Biological Process database. Plot based in the results found in the sheet 8 (S.Table.8-Hypergeometric-GOBP) of supplementary excel File S1.

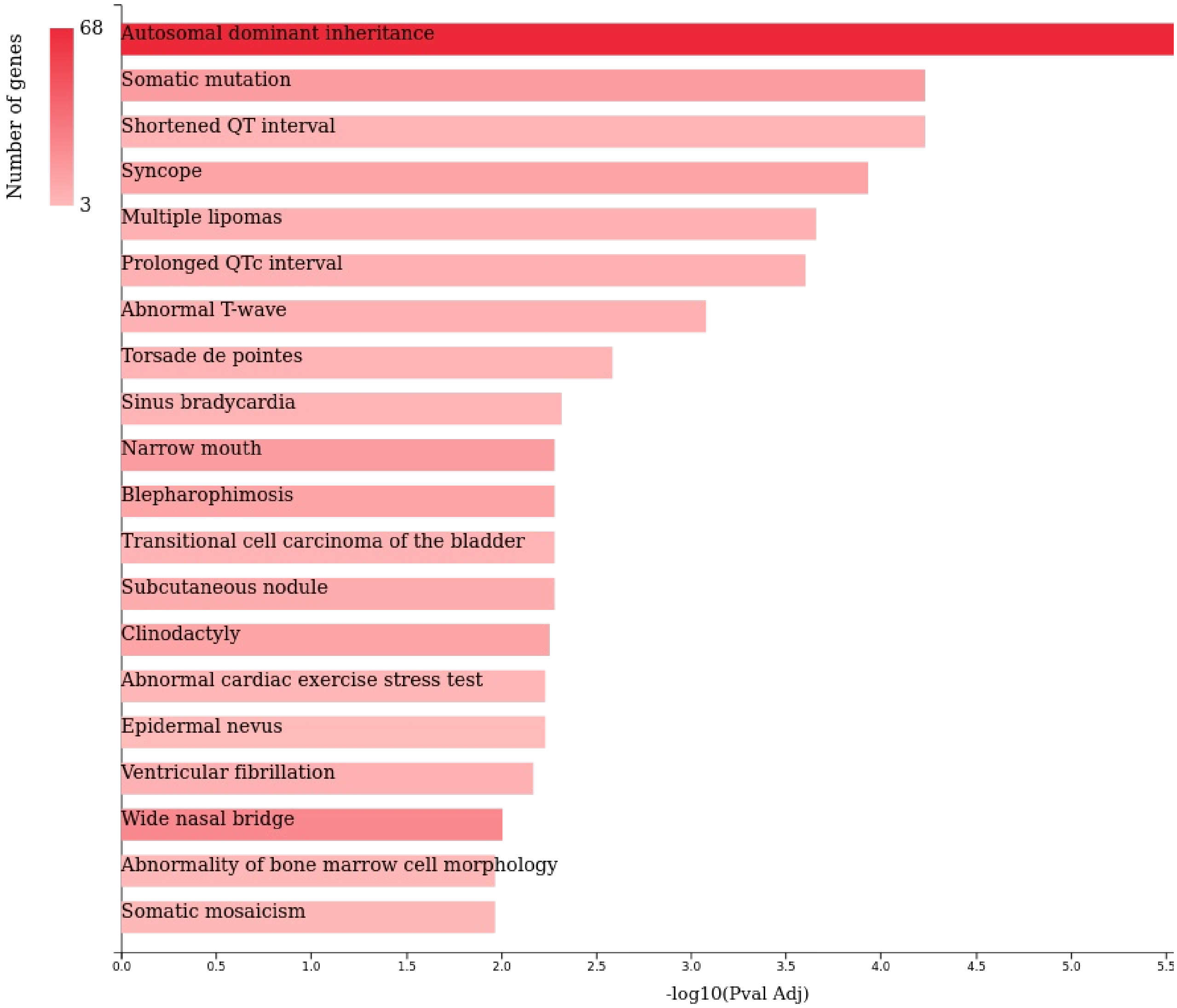

Figure S18. Bars chart with the top 20 terms found with the standard Hypergeometric approach and the Human Phenotype Ontology database. Plot based in the results found in the sheet 9 (S.Table.9-Hypergeometric-HPO) of supplementary excel File S1.

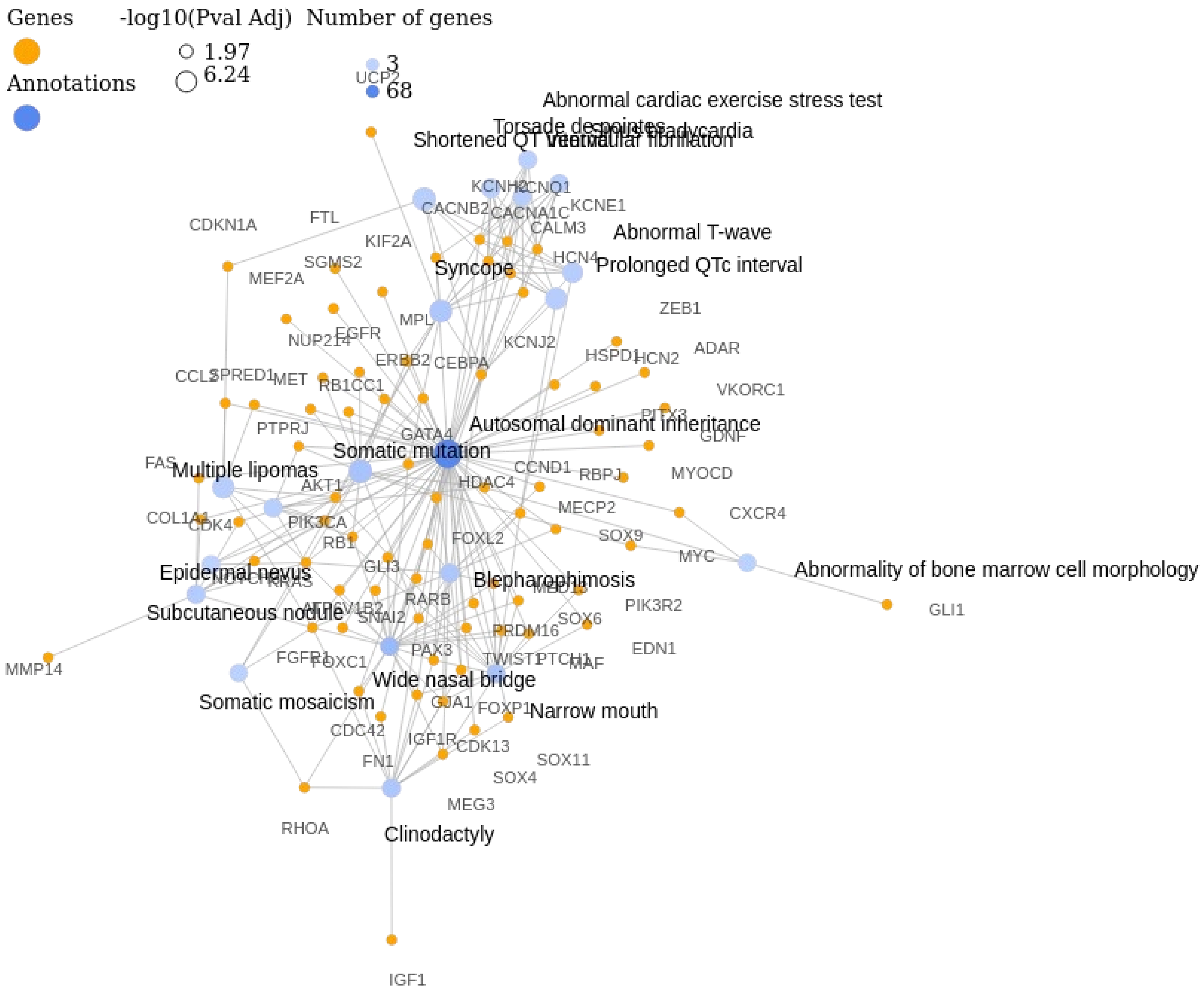

Figure S19. Network plot of gene and annotations of the top 20 terms found with the standard Hypergeometric approach and Human Phenotype Ontology database. Plot based in the results found in the sheet 9 (S.Table.9-Hypergeometric-HPO) of supplementary excel File S1.

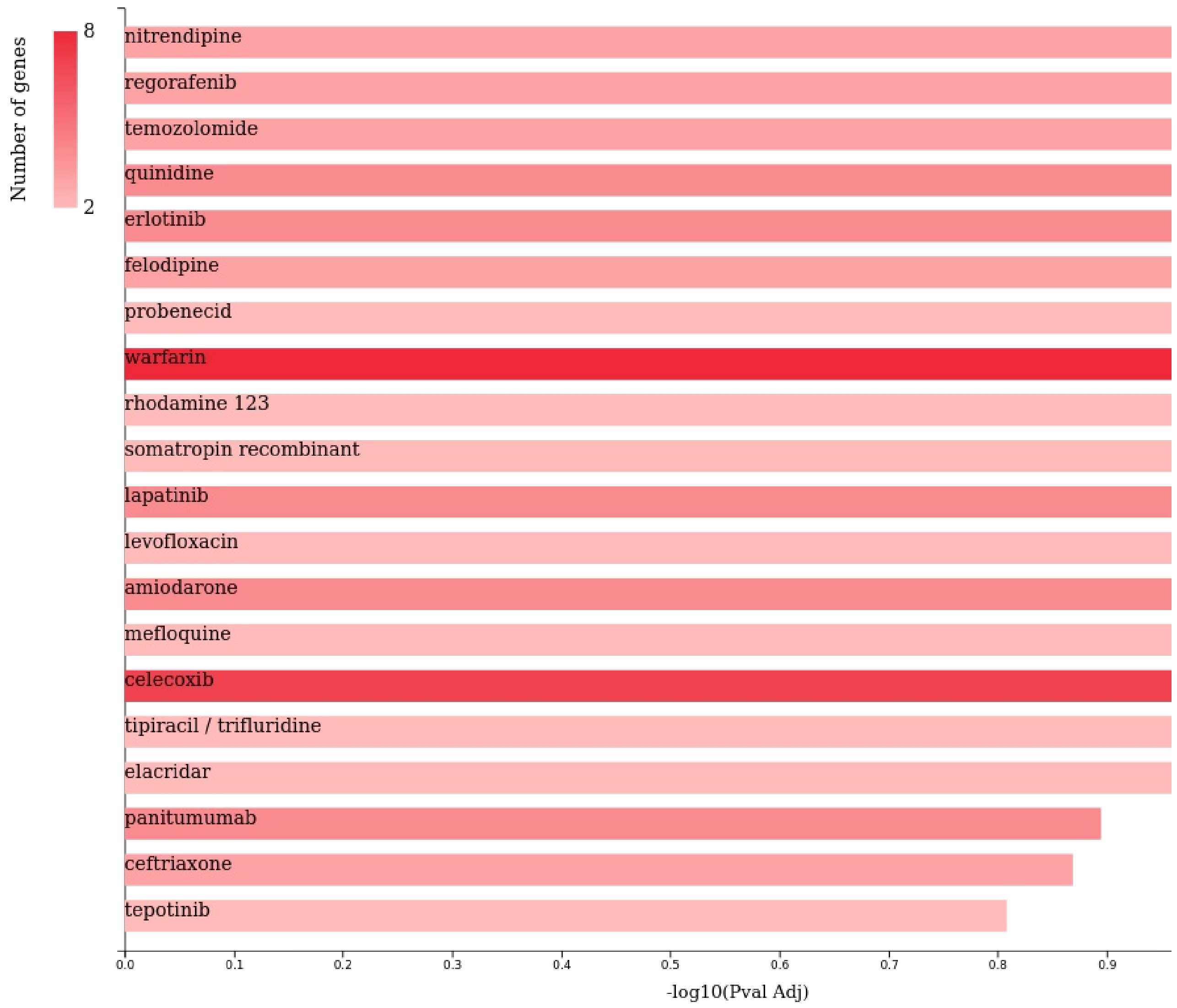

Figure S20. Bars chart with the top 20 terms found with the standard Hypergeometric approach and the PharmGKB database. Plot based in the results found in the sheet 10 (S.Table.10-Hypergeometric-PharmGKB) of supplementary excel File S1.

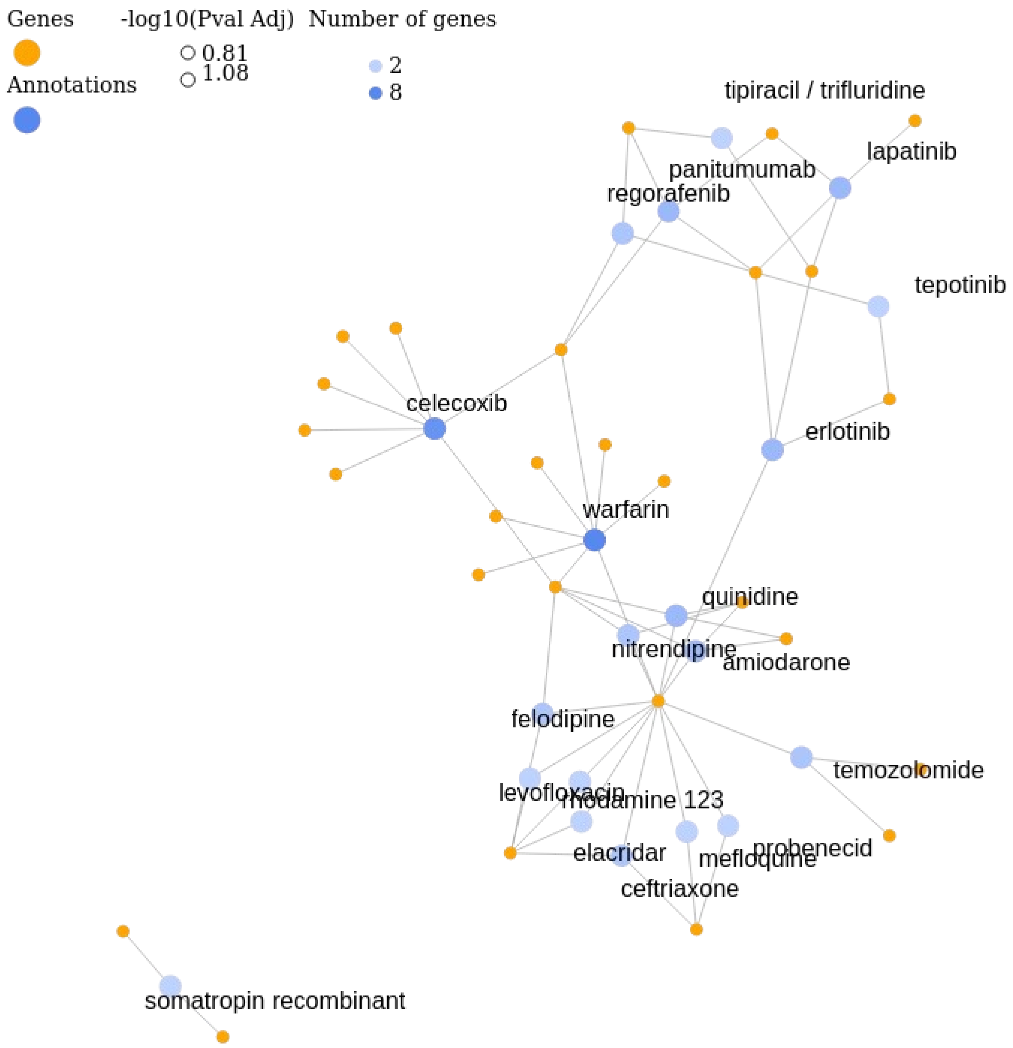

Figure S21. Network plot of gene and annotations of the top 20 terms found with the standard Hypergeometric approach with PharmGKB database. Plot based in the results found in the sheet 10 (S.Table.10-Hypergeometric-PharmGKB) of supplementary excel File S1.
